# Effects of Hytallzen™ on Growth-Related Parameters in Growing Male Rats

**DOI:** 10.3390/nu18132129

**Published:** 2026-07-01

**Authors:** Yujin Kim, Jinyoung Maeng, Ji Hye Yoon, Tae-Joon Kim, Junhyuk Choi, Sang Ho Lee, Young Hwa Park, Sangeun Im, Sung-Su Kim, Sungho Maeng, Jihwan Shin

**Affiliations:** 1 College of East-West Medical Science, Kyung Hee University, Yongin 17104, Republic of Koreasangho.lee@chamc.co.kr (S.H.L.);; 2 Department of Medical Device Industry, Seoul National University College of Medicine, 103 Daehak-ro, Jongno-gu, Seoul 03080, Republic of Korea; 3 College of Life Sciences and Biotechnology, Korea University, 145 Anam-ro, Seongbuk-gu, Seoul 02841, Republic of Korea; 4 College of Pharmacy, Dongduk Women’s University, Seoul 02748, Republic of Korea

**Keywords:** growth, Artemisia, Thymus, IGF-1

## Abstract

**Background/Objectives:** Hytallzen™ is a natural combined extract of Artemisia princeps and Thymus vulgaris. The present study evaluated its growth-promoting effects during the rapid growth period in male Sprague–Dawley rats. **Methods:** Hytallzen™ (150 mg/kg/day) was orally administered for 9 weeks beginning at postnatal week 3. Growth-related parameters, including body weight, body length, food intake, serum insulin-like growth factor-1 (IGF-1), and tibia length and weight, were assessed. **Results:** Hytallzen™ significantly increased body weight, body length, food intake, tibia length, and tibia weight compared with controls. In addition, serum IGF-1 concentration was significantly elevated at week 6 and showed positive correlations with final body weight, body length, tibia length, and tibia weight. **Conclusions:** These findings suggest that Hytallzen™ is associated with favorable changes in growth-related parameters and serum IGF-1 levels in healthy growing rats, supporting its potential as a growth-supportive material under normal physiological growth conditions.

## 1. Introduction

Physical growth during childhood and adolescence is a major determinant of lifelong health and quality of life [1]. Normal growth is regulated by multiple factors, including nutrition, genetics, endocrine signals, and environmental influences [2]. Among these, chronic stress and inflammation may disrupt endocrine homeostasis and tissue development, thereby impairing normal growth [3].

Hytallzen™ is a natural combined extract of Artemisia princeps and Thymus vulgaris. The Hytallzen™ material used in this study was supplied by Famenity Co., Ltd. (Uiwang-si, Republic of Korea) and was produced from source botanicals cultivated and processed under Famenity’s proprietary procedures. Because the biological activity of botanical materials may vary substantially depending on cultivation conditions, raw material control, extraction, fractionation, and purification processes, equivalent efficacy cannot be assumed for products derived from the same plant species by different manufacturing processes. In this context, the findings of the present study should be interpreted as applying specifically to the Hytallzen™ material evaluated herein.

A. princeps has long been used as a medicinal and edible plant in East Asia and is known for its anti-inflammatory, antioxidant, antimicrobial, and metabolic regulatory properties [4].

In addition, previous studies have shown that *A. princeps* attenuates bone loss and preserves trabecular and cortical bone microarchitecture in ovariectomized rats, suggesting its potential relevance to skeletal health [5]. Likewise, *T. vulgaris* is a widely used culinary and medicinal herb rich in polyphenols and volatile phytochemicals, including rosmarinic acid-related compounds [6]. It has been reported to exert antioxidant and anti-inflammatory effects and is recognized as a biologically active edible herb [7]. Although *T. vulgaris* has documented pharmacological properties, direct evidence for its growth-promoting effect remains limited, and available safety data appear to depend on the formulation used [8].

Several natural products have been investigated for height growth or longitudinal bone growth. The astragalus-based standardized mixture HT042 has shown both clinical and preclinical evidence of increasing height gain and modulating the GH(growth hormone)/IGF-1 axis [9]. Fermented oyster extract has also demonstrated clinical efficacy in promoting height growth in children, with possible involvement of IGFBP-3 (insulin-like growth factor binding protein-3)-related mechanisms [10]. In addition, a mixture of *Humulus japonicus* increased femur and tibia length, growth plate activity, and serum IGF-1/IGFBP-3 levels in normal rats [11]. Collectively, these studies suggest that natural growth-supportive materials may promote longitudinal growth through endocrine and local growth-regulatory pathways.

Based on these findings, we hypothesized that Hytallzen™ may support growth during the early growth period by providing a physiological environment favorable for longitudinal growth rather than acting as a direct growth hormone-like stimulant. Therefore, the present study investigated the effects of oral administration of Hytallzen™ on body weight, body length, food intake, serum IGF-1 concentration, and tibia growth in normally growing rats.

Hytallzen™ is being developed as a growth-supportive functional material intended to support normal growth and development rather than as a pharmaceutical growth-promoting agent. Therefore, the present study was designed to evaluate the effects of Hytallzen™ on growth-related parameters, including body weight, body length, food intake, serum IGF-1 concentration, and tibia growth, in normally growing rats under physiological growth conditions.

## 2. Materials and Methods

### 2.1. Plant Material and Extraction

Hytallzen™ is a combined extract of *Artemisia princeps* and *Thymus vulgaris*. The study material was provided by Famenity Co., Ltd. and was manufactured using Famenity’s proprietary cultivation, extraction, fractionation, and purification procedures. Accordingly, although the botanical sources are the same at the species level, the physicochemical characteristics and biological activity of the final material may differ depending on the source material control and manufacturing process. Therefore, the efficacy observed in the present study should not be generalized to all extracts prepared from *Artemisia princeps* and *Thymus vulgaris*, but rather should be regarded as specific to the proprietary Hytallzen™ preparation evaluated in this work. As the product profile is already publicly available, the present manuscript focuses on its biological evaluation rather than repeating the commercial profile in detail. In the chromatographic analysis used for material characterization, rosmarinic acid was used as a representative marker compound for quality control of Hytallzen™. Representative HPLC chromatograms and analytical validation data are provided in Supplementary Figure S1.

### 2.2. Animals and Experimental Design

**Figure 1:**
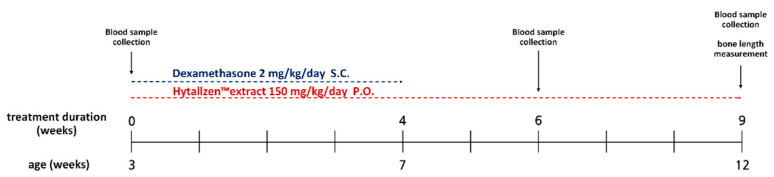
Schematic diagram of the experimental procedure. Hytallzen™ extract was administered orally from postnatal week 3 to 12. Body weight, body length, and food intake were measured weekly, and blood samples were collected at postnatal weeks 3, 9, and 12. n = 7/group.

Two-week-old male SD rats were obtained from Daehan Bio Link Co., Ltd. (Eumseong, Republic of Korea). Animals were pair-housed (two rats per cage) in polycarbonate cages under controlled environmental conditions (24 ± 1 °C, 55 ± 5% relative humidity) with a 12 h light/dark cycle (lights on at 6:00 and off at 18:00) and free access to standard chow and water. Cages were provided with paper bedding and environmental enrichment. Food intake was measured on a cage basis, and the average food intake per animal was calculated by dividing the total food consumption per cage by the number of animals housed in each cage. Each treatment group initially consisted of eight rats housed in pairs (two rats per cage). One animal per group was excluded during the experimental period; therefore, data from seven animals per group were included in the final analyses. After a one-week acclimatization period, rats were randomly divided into 2 groups (n = 7 per group): vehicle, distilled water p.o. for 9 weeks; and Hytallzen™, *Artemisia princeps* + *Thymus vulgaris* extract 150 mg/kg/day p.o. for 9 weeks (Figure 1). The dose of Hytallzen™ (150 mg/kg/day) was selected based on preliminary efficacy evaluation. The 9-week intervention period was selected to encompass the active growth phase from early postnatal development to young adulthood and to allow evaluation of longitudinal changes in growth-related parameters. Male rats were used to reduce variability associated with sex-dependent growth patterns during the developmental period. Healthy growing rats were selected because the primary objective of the present study was to evaluate the effects of Hytallzen™ on growth-related parameters under normal physiological growth conditions. All animal studies were conducted in accordance with the NIH Guide for the Care and Use of Laboratory Animals, and the protocols were approved by the Institutional Animal Care and Use Committee of Kyung Hee University (KHGASP-24-630).

### 2.3. Body Weight, Body Length, and Food Intake Measurement

Body weight was measured weekly from experimental week 0 to 9 (postnatal week 3 to 12) using an electronic scale. Weekly food intake was calculated by subtracting the weight of the remaining feed from the weight of the feed provided in the previous week. Spilled feed on the cage floor was recovered and included in the measurement.

Body length was measured immediately after body weight and food intake assessment. Each rat was individually placed in an induction chamber and exposed to 3% isoflurane in oxygen for approximately 1 min to minimize animal movement and relieve muscle tension during the measurement. The animals were placed in the prone position on a flat surface, and a ruler measuring 30 cm in length with a minimum graduation of 1 mm was used to measure the length from the tip of the nose to the tip of the sacrum. The head was gently stabilized with one hand, and the other hand was gently applied along the spine to straighten the trunk. The tail was excluded from the body length measurement. To minimize measurement error, the same experimenter measured each animal at least twice and repeated the measurement if the difference between the two measurements was greater than 3 mm. The final body length was determined when consistent results were obtained. After anesthesia, the animals were monitored for recovery and returned to their home cage.

### 2.4. Serum IGF-1 Measurement

Blood samples were collected from the tail vein at weeks 0, 6, and 9 after the start of Hytallzen™ administration. The collected blood was allowed to clot for 20 min at room temperature and then centrifuged at 3000 rpm for 20 min at 4 °C to separate the serum. The separated serum was transferred into 1.5 mL tubes and stored at −80 °C until analysis.

Serum IGF-1 concentrations were measured using an ELISA kit (Rat/Mouse IGF-1 ELISA Kit, Cat. No. ERLIF1, Thermo Fisher Scientific, Waltham, MA, USA) according to the manufacturer’s protocol. Briefly, serum samples were diluted 1:1000 in the sample diluent buffer. The standard curve range was 10–10,000 pg/mL, and the limit of detection was 30 pg/mL. The intra-assay coefficient of variation was <10%, and the inter-assay coefficient of variation was <12%. Absorbance at 450 nm was measured using a microplate reader (VICTOR X4, PerkinElmer, Shelton, CT, USA), and the IGF-1 concentration was calculated from the standard curve using four-parameter logistic regression. All samples were analyzed in duplicate.

### 2.5. Measurement of Tibia Length

After 9 weeks of treatment, rats were deeply anesthetized with ether in an induction chamber under a fume hood until loss of reflex responses was confirmed by toe or tail pinch. Blood samples were collected by abdominal incision under deep anesthesia, and the animals were euthanized by exsanguination. The pelvis and legs were then separated at the hip joint. The femur and tibia were then separated at the patella. Muscle, fat, growth plate, and cartilage attached to the bones were removed using forceps and scissors. The separated bones were dried in a 60 °C dry oven for 3 days.

The length of each bone was initially measured using a ruler with a minimum graduation of 1 mm and a total length of 30 cm. The images taken during the initial measurement were analyzed using ImageJ software (version 1.59g, National Institutes of Health, Bethesda, MD, USA). After calibrating the pixels in the images to millimeters based on the ruler graduations, the lengths of the left and right tibias were measured in decimal units. Tibia length was measured from the proximal end to the distal end.

### 2.6. Statistical Analysis

Data were expressed as mean ± standard error of the mean (SEM). Statistical analysis was performed using repeated-measures ANOVA followed by Tukey’s post hoc test using SPSS 22 (SPSS Inc., Chicago, IL, USA). A *p*-value below 0.05 was considered statistically significant.

## 3. Results

### 3.1. Hytallzen™ Increases Body Weight, Body Length, and Food Intake in Growing Rats

**Figure 2:**
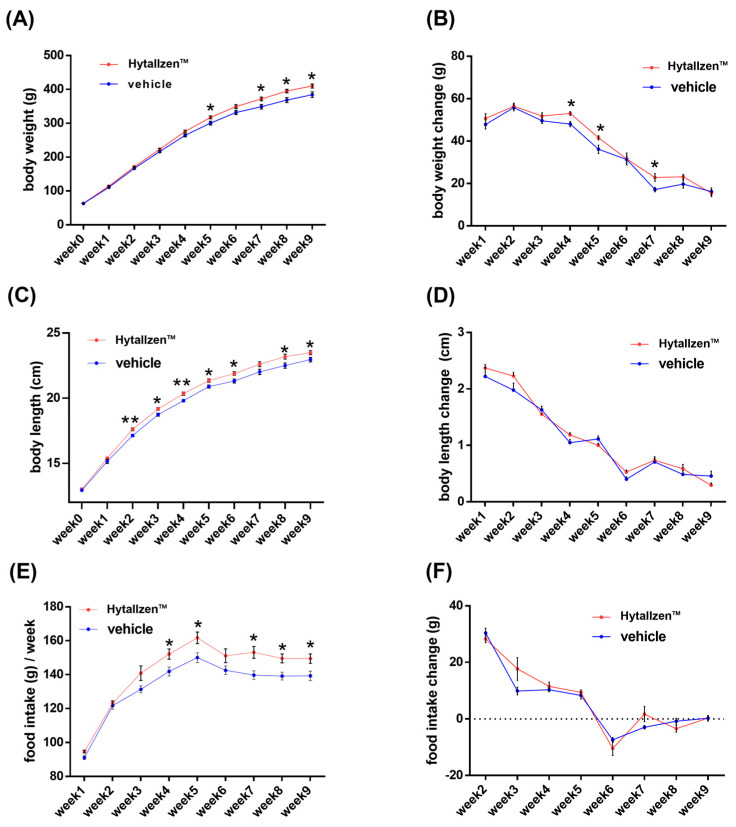
Hytallzen™ increases body weight, body length, and food intake in growing rats. (**A**) Weekly body weight during the experimental period. Time effect (within-subjects): F(9,108) = 3371, *p* < 0.001; group effect (between-subjects): F(1,12) = 5.8, *p* = 0.030; time × group interaction: F(9,108) = 5.7, *p* < 0.001. Post hoc test (vehicle vs. Hytallzen™): week 5, *p* = 0.034; week 7, *p* = 0.024; week 8, *p* = 0.013; week 9, *p* = 0.026; all other comparisons were not significant. (**B**) Weekly body weight gain. Time effect: F(8,96) = 196, *p* < 0.001; group effect: F(1,12) = 5.8, *p* = 0.033; time × group interaction: F(8,96) = 1.12, *p* = 0.350. Post hoc test (vehicle vs. Hytallzen™): week 4, *p* = 0.009; week 5, *p* = 0.024; week 7, *p* = 0.018; all other comparisons were not significant. (**C**) Weekly body length during the experimental period. Time effect: F(9,108) = 4622, *p* < 0.001; group effect: F(1,12) = 7.3, *p* = 0.020; time × group interaction: F(9,108) = 3.2, *p* = 0.002. Post hoc test (vehicle vs. Hytallzen™): week 2, *p* = 0.034; week 3, *p* = 0.014; week 4, *p* = 0.003; week 5, *p* = 0.022; week 6, *p* = 0.016; week 7, *p* = 0.054; week 8, *p* = 0.025; week 9, *p* = 0.038; all other comparisons were not significant. (**D**) Weekly body length gain. Time effect: F(8,96) = 177, *p* < 0.001; group effect: F(1,12) = 4.5, *p* = 0.055; time × group interaction: F(8,96) = 1.7, *p* = 0.107. (**E**) Weekly food intake during the experimental period. Time effect: F(9,96) = 270, *p* < 0.001; group effect: F(1,12) = 7.4, *p* = 0.018; time × group interaction: F(9,96) = 2.8, *p* = 0.007. Post hoc test (vehicle vs. Hytallzen™): week 1, *p* = 0.020; week 2, *p* = 0.630; week 3, *p* = 0.068; week 4, *p* = 0.024; week 5, *p* = 0.023; week 6, *p* = 0.097; week 7, *p* = 0.009; week 8, *p* = 0.011; week 9, *p* = 0.022. (**F**) Weekly change in food intake. Time effect: F(7,84) = 89, *p* < 0.001; group effect: F(1,12) = 3.6, *p* = 0.080; time × group interaction: F(7,84) = 2.3, *p* = 0.037. Post hoc test (vehicle vs. Hytallzen™): all comparisons were not significant. All data are presented as mean ± SEM. n = 7 per group. Vehicle, oral administration of distilled water for 9 weeks; Hytallzen™, oral administration of Hytallzen™ at 150 mg/kg for 9 weeks. * *p* < 0.05, ** *p* < 0.01 vs. vehicle. Data were analyzed by repeated-measures ANOVA followed by Tukey’s post hoc test.

Three-week-old male rats were orally administered Hytallzen™ or vehicle for 9 weeks. Statistical analysis revealed significant treatment-related effects on body weight, body length, and food intake throughout the experimental period. The Hytallzen™-treated group showed significantly greater body weight than the vehicle group at weeks 5, 7, 8, and 9 (Figure 2A). Weekly body weight gain was also significantly higher in the Hytallzen™ group at weeks 4, 5, and 7 (Figure 2B). Body length was significantly greater in the Hytallzen™ group from the second week of administration (Figure 2C). This difference appears to have resulted from a modest but cumulative increase in body length during weeks 1 and 2, although the weekly increments did not reach statistical significance (Figure 2D). Food intake was significantly higher in the Hytallzen™ group after week 4 (Figure 2E). The weekly change in food intake gradually decreased with age and remained relatively stable after week 7 (Figure 2F).

### 3.2. Hytallzen™ Increased Serum IGF-1 Concentration in Growing Rats

**Figure 3:**
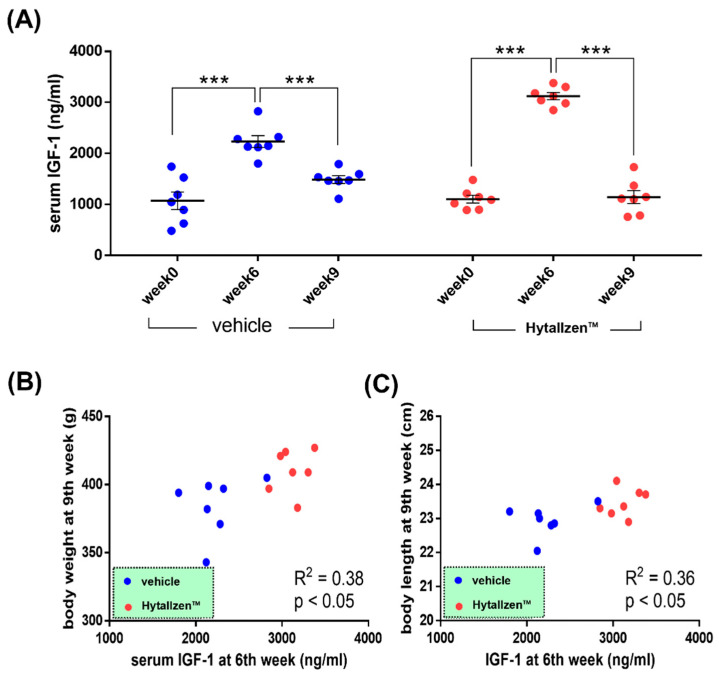
Effects of Hytallzen™ on serum IGF-1 levels in growing rats. (**A**) Serum IGF-1 concentrations in the vehicle and Hytallzen™-treated groups at weeks 0, 6, and 9. Time effect (within-subjects): F(2,24) = 110, *p* < 0.001; group effect (between-subjects): F(1,12) = 4.83, *p* = 0.048; time × group interaction: F(2,24) = 15, *p* < 0.001. (**B**) Pearson’s correlation between serum IGF-1 concentration at week 6 and body weight at week 9. (**C**) Pearson’s correlation between serum IGF-1 concentration at week 6 and body length at week 9. All data are presented as mean ± SEM. n = 7 per group. Vehicle, oral administration of distilled water for 9 weeks; Hytallzen™, oral administration of Hytallzen™ at 150 mg/kg for 9 weeks. *** *p* < 0.001. Data were analyzed by repeated-measures ANOVA followed by Tukey’s post hoc test.

During Hytallzen™ treatment, serum IGF-1 concentrations were measured at week 0 (3 weeks of age), week 6 (9 weeks of age), and week 9 (12 weeks of age) (Figure 3). Statistical analysis revealed significant treatment-related effects on serum IGF-1 concentration during the experimental period. In both groups, serum IGF-1 concentration increased at week 6 compared with week 0. At week 6, the Hytallzen™-treated group showed a higher serum IGF-1 concentration than the vehicle group. At week 9, serum IGF-1 concentration decreased compared with week 6. Higher serum IGF-1 concentration at week 6 was positively correlated with final body weight and body length at week 9 (Figure 3B,C). These results indicate that Hytallzen™ increased serum IGF-1 concentration during the active growth period and that this increase was associated with subsequent somatic growth.

### 3.3. Hytallzen™ Increased Tibia Length and Weight

**Figure 4:**
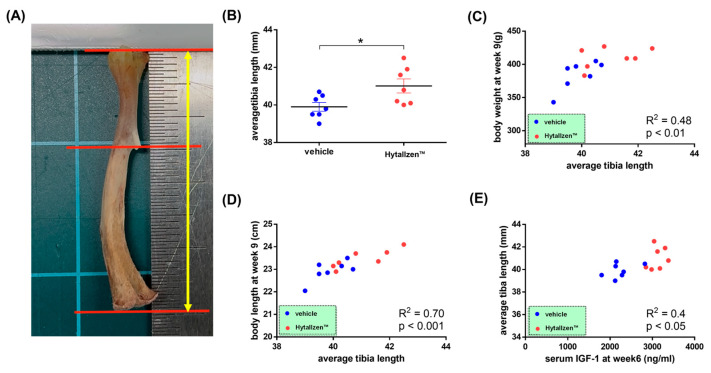
Hytallzen™ increases tibia length in growing rats. (**A**) Representative image showing tibia length measurement (yellow arrow). (**B**) Mean tibia length, calculated as the average of the left and right tibias. (**C**–**E**) Pearson’s correlations of mean tibia length with body weight at week 9 (**C**), body length at week 9 (**D**), and serum IGF-1 concentration at week 6 (**E**). All data are presented as mean ± SEM. n = 7 per group. Vehicle, oral administration of distilled water from week 0 to week 9; Hytallzen™, oral administration of Hytallzen™ at 150 mg/kg from week 0 to week 9. * *p* < 0.05 vs. vehicle group. Statistical significance in (**B**) was analyzed using an unpaired *t*-test.

**Figure 5:**
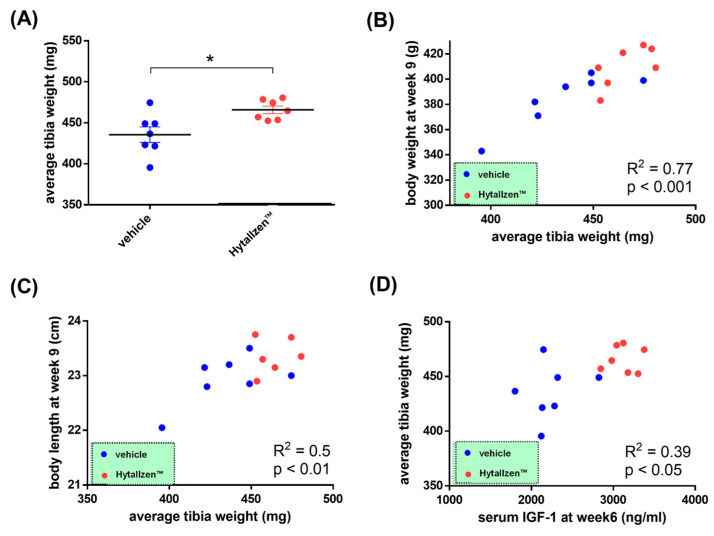
Hytallzen™ increases tibia weight in growing rats. (**A**) Mean tibia weight, calculated as the average of the left and right tibias. (**B**–**D**) Pearson’s correlations of mean tibia weight with body weight at week 9 (**B**), body length at week 9 (**C**), and serum IGF-1 concentration at week 6 (**D**). All data are presented as mean ± SEM. n = 7 per group. Vehicle, oral administration of distilled water from week 0 to week 9; Hytallzen™, oral administration of Hytallzen™ at 150 mg/kg from week 0 to week 9. * *p* < 0.05 vs. vehicle group. Statistical significance in (**A**) was analyzed using an unpaired *t*-test.

Tibia length was assessed after 9 weeks of Hytallzen™ administration (Figure 4A). The mean tibia length, calculated as the average of the left and right tibias, was significantly greater in the Hytallzen™ group than in the vehicle group (Figure 4B). In addition, mean tibia length was positively correlated with body weight and body length at week 9 (Figure 4C,D) and with serum IGF-1 concentration at week 6 (Figure 4E). Similarly, tibia weight was significantly higher in the Hytallzen™ group than in the vehicle group (Figure 5A). Mean tibia weight was positively correlated with body weight and body length at week 9 (Figure 5B,C), as well as with serum IGF-1 concentration at week 6 (Figure 5D).

## 4. Discussion

Previous studies have suggested that *Artemisia princeps* and *Thymus vulgaris* may provide a physiological background favorable for skeletal health and growth-related processes. *Artemisia princeps* has been reported to exhibit anti-inflammatory and antioxidant activities, and its extract attenuated bone mineral density loss in ovariectomized rats [5]. *Thymus vulgaris* is also recognized as a biologically active edible herb with antioxidant and anti-inflammatory properties [12]. Based on these findings, Hytallzen™, a combined extract of *Artemisia princeps* and *Thymus vulgaris*, was considered a promising candidate for promoting growth during the early growth period. An important point in interpreting the present findings is that the tested material was not a generic botanical mixture, but rather the proprietary Hytallzen™ preparation supplied by Famenity Co., Ltd. Because cultivation conditions, source material standardization, extraction parameters, and fractionation/purification steps can substantially influence the chemical composition and biological activity of botanical preparations, the growth-promoting effects observed in this study should be understood as being attributable to the specific Hytallzen™ material used in this work rather than to all preparations derived from the same plant species.

This study demonstrates that oral administration of Hytallzen™ increases body weight, body length, food intake, serum IGF-1 concentration, tibia length, and tibia weight in rats during the growth period.

### 4.1. Growth-Promoting Effect of Hytallzen™ in Normal Rats

In this study, when male rats were fed Hytallzen™ starting at the third postnatal week, the Hytallzen™-treated group remained heavier after week 5 compared to non-treated rats. These weight differences resulted from greater weight gain in the Hytallzen™ group during weeks 4, 5, and 7. These findings suggest that increased body weight may require at least 4 weeks of Hytallzen™ administration to become clearly apparent.

On the other hand, body length remained greater in the Hytallzen™ group after 2 weeks of administration. Although the weekly increase in body length in weeks 1 and 2 was not significantly greater than that in the control group, this small difference accumulated over time and resulted in a longer body length. The weekly increase in body length remained similar after week 3; therefore, the increase in body length due to Hytallzen™ appears to be an early effect that emerged within 2 weeks of administration.

Food intake was maintained at a higher level in the Hytallzen™ group after week 4. Intake increased in both groups until week 5 and then remained relatively constant after week 7. However, the period with the greatest difference in food intake was the third week. In the Hytallzen™ group, body length began to increase after the second week, whereas food intake began to increase after the third week. This temporal pattern suggests that the initial increase in body length may not have been driven simply by increased nutrient intake, but rather by improved utilization of available nutrients for growth. In other words, Hytallzen™ may be associated with improved utilization of available nutrients during the growth period.

Meanwhile, body weight in the Hytallzen™ group increased compared to the control group after week 5, which appears to be associated with the increased food intake observed after week 4. In summary, body length growth due to Hytallzen™ was most evident in the second week of treatment and was then maintained thereafter. Food intake did not increase further during the second week when body length growth was accelerated. In contrast, weight gain in the Hytallzen™ group accelerated from week 5, after food intake had already increased. Taken together, these findings suggest that Hytallzen™ may be associated with early changes in body length and later increases in body weight as nutrient intake increases. Previous studies have demonstrated that nutritional status and endocrine factors coordinately regulate somatic growth during childhood and adolescence [1,2,3]. In particular, natural growth-supportive materials such as HT042 have been reported to modulate growth-related endocrine pathways, including circulating IGF-1 levels [9]. Therefore, the early increase in body length observed in the present study may reflect a physiological environment favorable for growth rather than a simple consequence of increased food intake. Nevertheless, the increased food intake observed in the Hytallzen™ group may have contributed, at least in part, to the observed growth-related outcomes and should be considered when interpreting the present findings.

### 4.2. Hytallzen™ and Serum IGF-1 Concentration

Serum IGF-1 concentration was measured before the start of the experiment (week 0) and at weeks 6 and 9. In the normal groups, with or without Hytallzen™ treatment, IGF-1 concentrations increased at week 6 compared with week 0 but decreased at week 9 compared with week 6. At week 6, the IGF-1 concentration was higher in the Hytallzen™ group than in the control group. Considering that the IGF-1 concentration at week 6 was correlated with final body weight and body length measured at week 9 (Figure 3B,C), the increased serum IGF-1 concentration in the Hytallzen™ group may be associated with the observed changes in body weight and body length. However, because the IGF-1 concentration in the Hytallzen™ group was lower than that in the control group at week 9, the growth-promoting effect of Hytallzen™ may be more relevant during an earlier developmental window rather than during later stages of growth. This interpretation is consistent with the observation that body length responded earlier than body weight and that the main growth-promoting effects were most evident during the early phase of administration.

Overall, these results suggest that Hytallzen™ administration was associated with increased serum IGF-1 concentration during the active early growth period. Previous studies have shown that growth-supportive natural products, including HT042 and fermented oyster extract, increase circulating IGF-1 levels and are associated with enhanced growth outcomes in both experimental animals and children [9,10]. Consistent with these reports, the present findings suggest that modulation of serum IGF-1 may represent one mechanism underlying the growth-related effects associated with Hytallzen™ administration.

### 4.3. Hytallzen™ on Tibia Length and Weight

The length and weight of the tibia measured at week 9 of the experiment (12 weeks of age) also increased more in the Hytallzen™-treated group. To examine whether the increase in tibia length and weight was valid as an indicator of growth, the correlations with body weight and body length were analyzed, and both tibia length and tibia weight were positively correlated with body weight and body length. The concentration of serum IGF-1 measured at week 6 also showed a correlation with tibia length and tibia weight.

In other words, higher IGF-1 concentrations were associated with longer and heavier tibias, which in turn were associated with increased body weight and body length. These findings suggest that the week 6 IGF-1 concentration may reflect a critical time point associated with active skeletal growth in this model. Therefore, the increase in tibia length and weight provides additional evidence that Hytallzen™ was associated not only with external body growth but also with longitudinal bone growth-related parameters during the normal growth period. Similar findings have been reported for other natural growth-supportive materials. HT042 has been reported to modulate circulating IGF-1 levels, whereas Humulus japonicus enhanced longitudinal bone growth together with increased serum IGF-1 levels [9,11]. The positive correlations observed between serum IGF-1 concentration and tibia growth parameters in the present study are in agreement with these previous observations.

### 4.4. General Tolerability

At the tested dose of 150 mg/kg/day, repeated oral administration of Hytallzen™ for 9 weeks was well tolerated in growing rats. During the study period, the general condition of the animals remained stable, and food intake was maintained without reduction. In addition, body weight, body length, and tibia-related growth parameters were preserved and increased in a physiologically consistent manner throughout the experimental period. These findings indicate that repeated oral administration of Hytallzen™ was well tolerated with respect to the endpoints assessed under the present experimental conditions.

Despite these findings, several limitations should be considered when interpreting the present results. The study was conducted in male rats using a single dose of Hytallzen™ and a vehicle-treated control group only. In addition, the findings were obtained in healthy growing rats under normal physiological conditions. Therefore, the observed effects should be interpreted within the context of the present experimental design and may not be directly extrapolated to growth-impaired conditions or pediatric populations.

## 5. Conclusions

This study provides evidence that Hytallzen™ is associated with increases in body weight, body length, and serum IGF-1 levels in young male rats. These effects appear to be most evident during the early postnatal growth period, and the growth-promoting effect on body length seems to emerge earlier than the effect on body weight. Furthermore, the positive correlations among serum IGF-1 concentration, body growth, and tibia growth suggest an association between Hytallzen™ administration and growth-related parameters during early development. Collectively, these findings support the potential of Hytallzen™ as a growth-supportive material under normal physiological growth conditions. Under the present study conditions, repeated oral administration of Hytallzen™ was well tolerated and was associated with stable growth and maintained food intake.

## Data Availability

The raw data supporting the conclusions of this article will be made available by the authors on request.

## Supplementary Materials

The following supporting information can be downloaded at: https://www.mdpi.com/article/10.3390/nu18132129/s1, Figure S1: Representative HPLC chromatograms and UV spectra of rosmarinic acid in Hytallzen™; Table S1: Growth-Related Parameters in Vehicle- and Hytallzen™-Treated Rats; Table S2: Serum IGF-1 Concentrations in Vehicle- and Hytallzen™-Treated Rats.

## Abbreviations

The following abbreviations are used in this manuscript:

IGF-1	insulin-like growth factor-1
IGFBP-3	insulin-like growth factor binding protein-3
GH	Growth hormone
SEM	Standard error of the mean
