# Supplementary material for: Effects of Hytallzen™ on Growth-Related Parameters in Growing Male Rats
Source: Nutrients. 2026 Jul 1;18(13):2129. doi: 10.3390/nu18132129 (PMC13364358; doi:10.3390/nu18132129)
Supplement: Supplementary file 1 [file nutrients-18-02129-s001.zip › nutrients-4337277-supplementary.pdf]

Supplementary Figure S1

Representative HPLC chromatograms and UV spectra of rosmarinic acid in Hytallzen™.

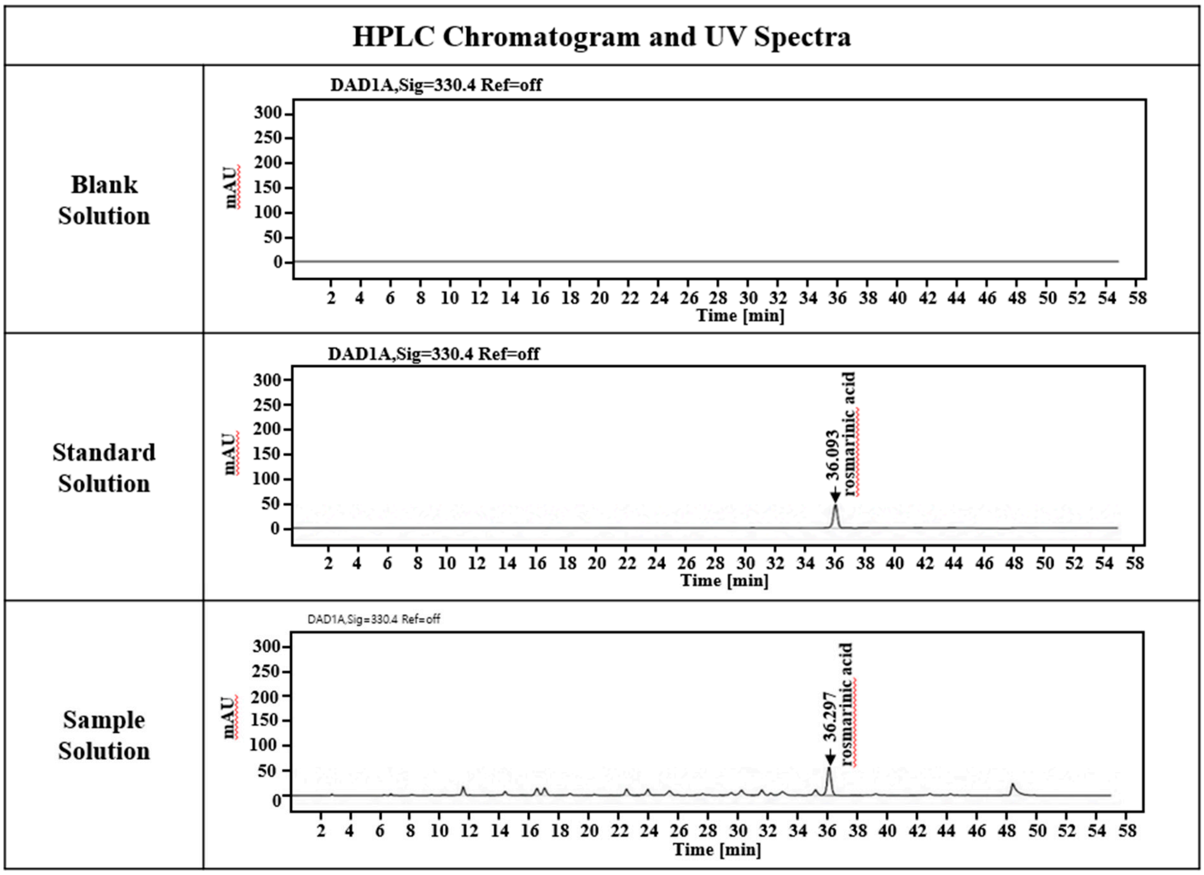


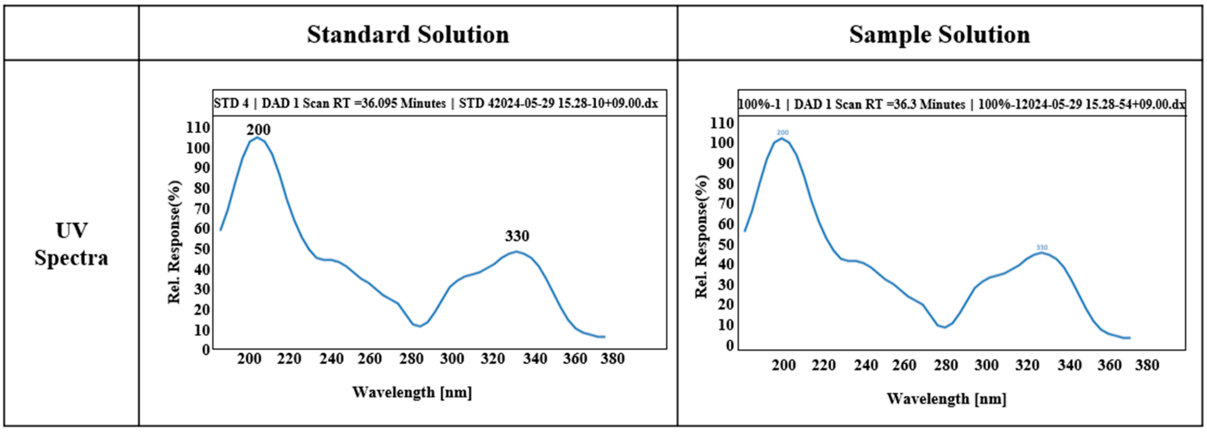

Supplementary Table S1.

Growth-Related Parameters in Vehicle- and Hytallzen™-Treated Rats

| Parameter | Vehicle | Hytallzen™ | % Change |
|---|---|---|---|
| Body weight (Week 9) (g) | 384.2 ± 7.9 | 409.8 ± 5.9 | +6.7% |
| Body length (Week 9) (cm) | 22.9 ± 0.17 | 23.46 ± 0.15 | +2.4% |
| Tibia weight (Week 9) (mg) | 871.1 ± 19.0 | 931.7 ± 8.5 | +7.0% |
| Tibia length (Week 9) (mm) | 39.9 ± 0.22 | 41.01 ± 0.37 | +2.8% |

Values are presented as mean ± SEM (n = 7 per group).

Supplementary Table S2.

Serum IGF-1 Concentrations in Vehicle- and Hytallzen™-Treated Rats

| Time | Vehicle (ng/mL) | Hytallzen™ (ng/mL) | % Change |
|---|---|---|---|
| Week 0 | 1074.1 ± 157.5 | 1105.9 ± 74.2 | +3.0% |
| Week 6 | 2232.5 ± 120.6 | 3121.4 ± 65.7 | +39.8% |
| Week 9 | 1453.4 ± 81.0 | 1144.7 ± 117.5 | -21.2% |

Values are presented as mean ± SEM (n = 7 per group).
